# Ultrasound-Enhanced Chemical Tissue Clearing of Large Specimens for 3D Microscopy: A Rapid and Accessible Method Using Standard Laboratory Equipment

**DOI:** 10.3390/mps9040119

**Published:** 2026-08-14

**Authors:** Klaus Becker, Seyed Meraaj Foroughipour, Massih Foroughipour, Karoline Maria Schwendt, Stefan H. Geyer, James Oakes-Klein, Christoph Fuchssteiner, Wolfgang J. Weninger, Eugenijus Kaniusas, Saiedeh Saghafi

**Affiliations:** 1 Meso-Aspheric Optics and LSFM, Institute of Solid State Electronics, Technische Universität Wien, 1060 Vienna, Austria; seyed.meraaj.foroughipour@univie.ac.at (S.M.F.); seyed.massih.foroughipour@univie.ac.at (M.F.); saiedeh.saghafi@tuwien.ac.at (S.S.); 2 Center for Anatomy and Cell Biology, Medical University of Vienna, 1090 Vienna, Austria; karoline.schwendt@meduniwien.ac.at (K.M.S.); stefan.geyer@meduniwien.ac.at (S.H.G.); christoph.fuchssteiner@meduniwien.ac.at (C.F.); wolfgang.weninger@meduniwien.ac.at (W.J.W.); 3 Department of Molecular Neurosciences, Center for Brain Research, Medical University Vienna, 1090 Vienna, Austria; james.oakes-klein@meduniwien.ac.at; 4 Institute of Biomedical Electronics, Technische Universität Wien, 1060 Vienna, Austria; eugenijus.kaniusas@tuwien.ac.at

**Keywords:** 3D imaging, chemical tissue clearing, dibenzyl ether, light sheet microscopy, refractive index matching, ultrasound

## Abstract

While the recently developed tissue clearing protocols pathoDISCO and activeDISCO significantly accelerate the clearing of large tissue specimens through active chemical dehydration using 2,2-dimethoxypropane, the final step of refractive index (RI) matching with viscous organic solvents as dibenzyl ether (DBE) remains restricted by slow passive diffusion. To overcome this bottleneck, we applied 40 kHz ultrasound using a standard, cost-effective laboratory bath to significantly enhance the diffusion kinetics of the clearing medium into large specimens. Our investigation on multi-centimeter-sized porcine muscle and human earlobe samples demonstrates that 40 kHz acoustic oscillations generated by a standard ultrasound cleaning device not only accelerate the clearing process but also yield superior and stable long-term optical transparency. We also tested 1 MHz high-frequency ultrasound but it offered no kinetic advantages and tended to induce tissue micro-fractures, an artifact we have not observed at 40 kHz at comparable energy levels. We therefore propose that standard 40 kHz ultrasound baths, ubiquitous in laboratories for cleaning purposes, represent an ideal and accessible tool for optimizing solvent-based tissue clearing.

## 1. Introduction

The comprehensive spatial analysis of biological systems, ranging from whole-brain neuronal projections to intricate neuronal and vascular networks, often necessitates three-dimensional (3D) visualization. This requirement has driven the development of chemical tissue clearing techniques, a suite of methods capable of rendering opaque biological samples optically transparent. When coupled with light sheet fluorescence microscopy (LSFM), these methods enable rapid, organ-wide imaging even at cellular resolution [1].

A fundamental objective of tissue clearing is to homogenize the refractive indices (RIs) of diverse tissue components, principally lipids, water, and proteins, to minimize light scattering within the tissue [2]. Current protocols generally fall into three main families: 1. aqueous-based methods utilizing hydrophilic reagents [3]; 2. hydrogel-based methods transforming the specimen into a tissue-hydrogel hybrid [4]; and 3. organic solvent-based protocols, which dehydrate and delipidate the sample before immersion in a high-RI organic solvent. This final family is renowned for its speed and high degree of transparency [5].

Despite this diverse toolbox, a universal limitation for clearing large samples as entire tumor biopsies or large brain samples remains: time. The primary bottleneck for nearly all protocols is the reliance on the slow, passive diffusion of reagents into dense tissue. To overcome this time barrier, active methods employing electrophoresis, pressure, or perfusion have been developed [6]. Sabdyusheva Litschauer et al. (2020) demonstrated for the first time that the slow, diffusion-limited tissue dehydration step, which typically utilizes a concentration gradient of alcohol or another water-miscible intermediate, can be replaced by an active, single-step dehydration procedure [7]. This approach utilizes the chemical hydrolysis of the acetal compound 2,2-dimethoxypropane (DMP) into acetone and methanol, thereby actively consuming the water in the specimen. Unlike passive dehydration protocols that rely on the slow, two-way passive diffusion of water out of the sample and solvents into the tissue matrix, DMP penetrates the cell membranes due to its fat-solving properties and chemically consumes the water molecules in situ [8]. By converting the trapped water directly from within, this reaction fundamentally bypasses the physical limits of Fickian diffusion [9]. This active chemical process enables the complete dehydration of exceptionally large biological samples, such as multiple centimeter-sized tumor biopsies (pathoDISCO) [7], or large human brain specimens (PathoDISCO-HE) [10] in a time range of just a few hours.

However, while active dehydration with DMP successfully accelerates the removal of water, a significant gap remains in accelerating the final RI-matching step. The penetration of relatively viscous clearing media like dibenzyl ether (DBE) is still governed by slow passive diffusion. While this is a minor problem for small samples such as mouse brains this becomes increasingly problematic as specimen volumes scale up to multiple cubic centimeters.

While the application of acoustic energy is well established for enhancing diffusion in tissue fixation and immunostaining [11,12,13], its potential has only recently been explored for tissue clearing. Recent studies have demonstrated that 40 kHz ultrasound can successfully accelerate both aqueous-based [14] and organic-solvent-based [15] clearing protocols in small rodent tissues. Nevertheless, its specific efficacy in overcoming the final diffusion bottleneck of highly viscous solvents like DBE, particularly following rapid active chemical dehydration in exceptionally large, multi-centimeter-sized tissue specimens, remains uncharacterized.

In this study, we investigated the effects of 40 kHz and 1 MHz ultrasound on tissue clearing to further accelerate our fast-clearing pipeline, using DMP for ultrafast tissue dehydration [7,10] and DBE for subsequent RI matching. We assessed the impact of the applied acoustic oscillations on clearing speed and final optical transparency to establish an optimized, accessible protocol that successfully mitigates the final temporal bottleneck in large-scale tissue clearing.

## 2. Materials and Methods

**Figure 1:**
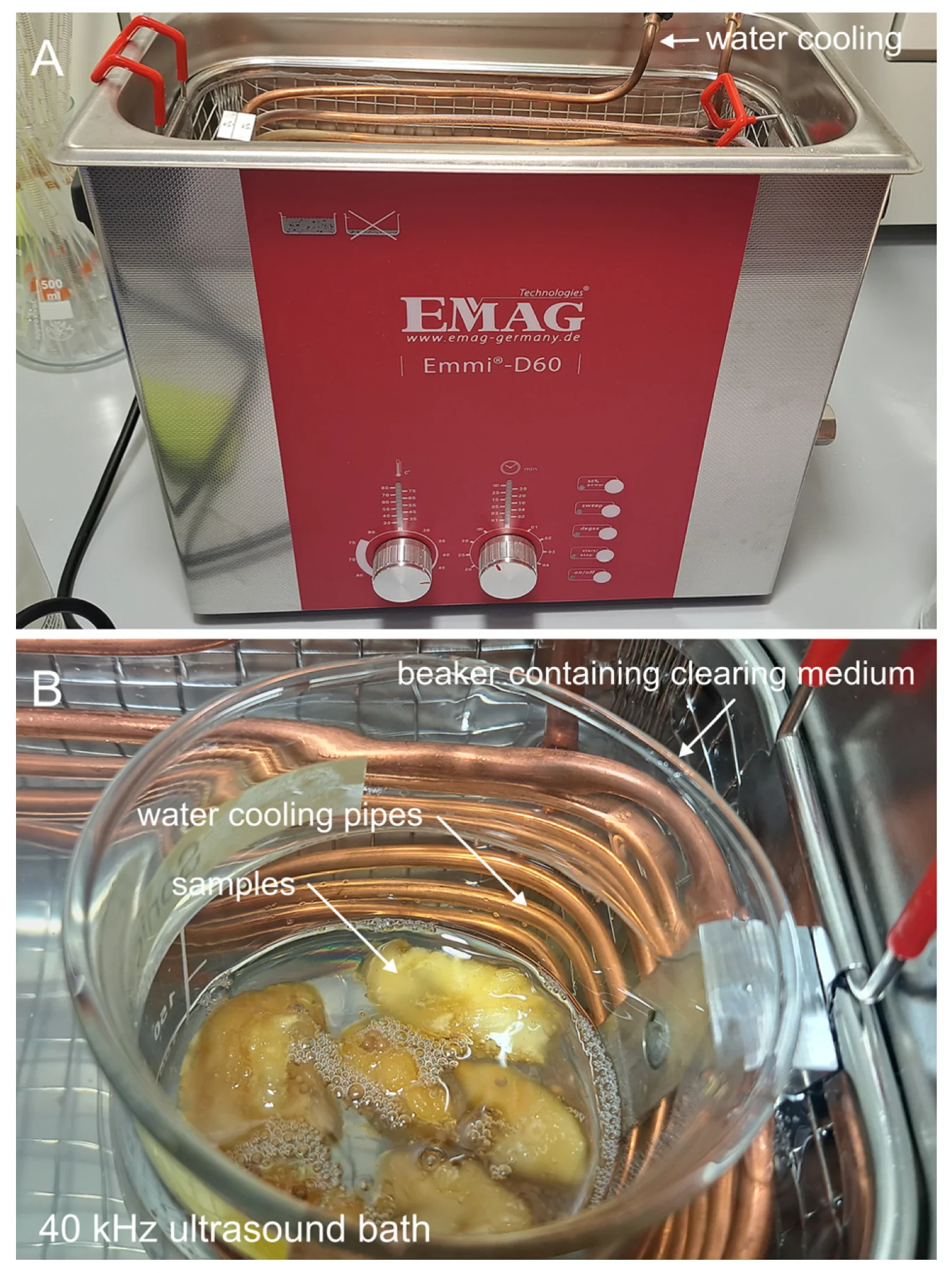
**Experimental setup for 40 kHz ultrasound-assisted tissue clearing.** (**A**) The standard 40 kHz ultrasound cleaning bath (Emmi-D60, EMAG) used for the experiments. (**B**) View of the water tank: A beaker containing the tissue samples and clearing medium (DBE) is immersed in the water bath. A custom-made spiral copper tube circulates cold tap water to remove the heat generated by the transmitted ultrasound energy, maintaining the temperature at approx. 20 °C.

**Ultrasound Generation and Temperature Control:** Ultrasound waves of 40 kHz frequency were generated using an Emmi-D60 ultrasound bath (EMAG, Mörfelden-Walldorf, Germany) delivering a maximal power of 320 W (Figure 1A). External cooling was required to prevent excessive temperature increases of over 50 °C that could damage the samples. Cooling was achieved using cold tap water flowing through a spiral copper tube of 5 mm diameter for the 40 kHz device (Figure 1B). By adjusting the water flow, we kept the temperature at a stable equilibrium of 20 °C during the entire clearing process.

**Figure 2:**
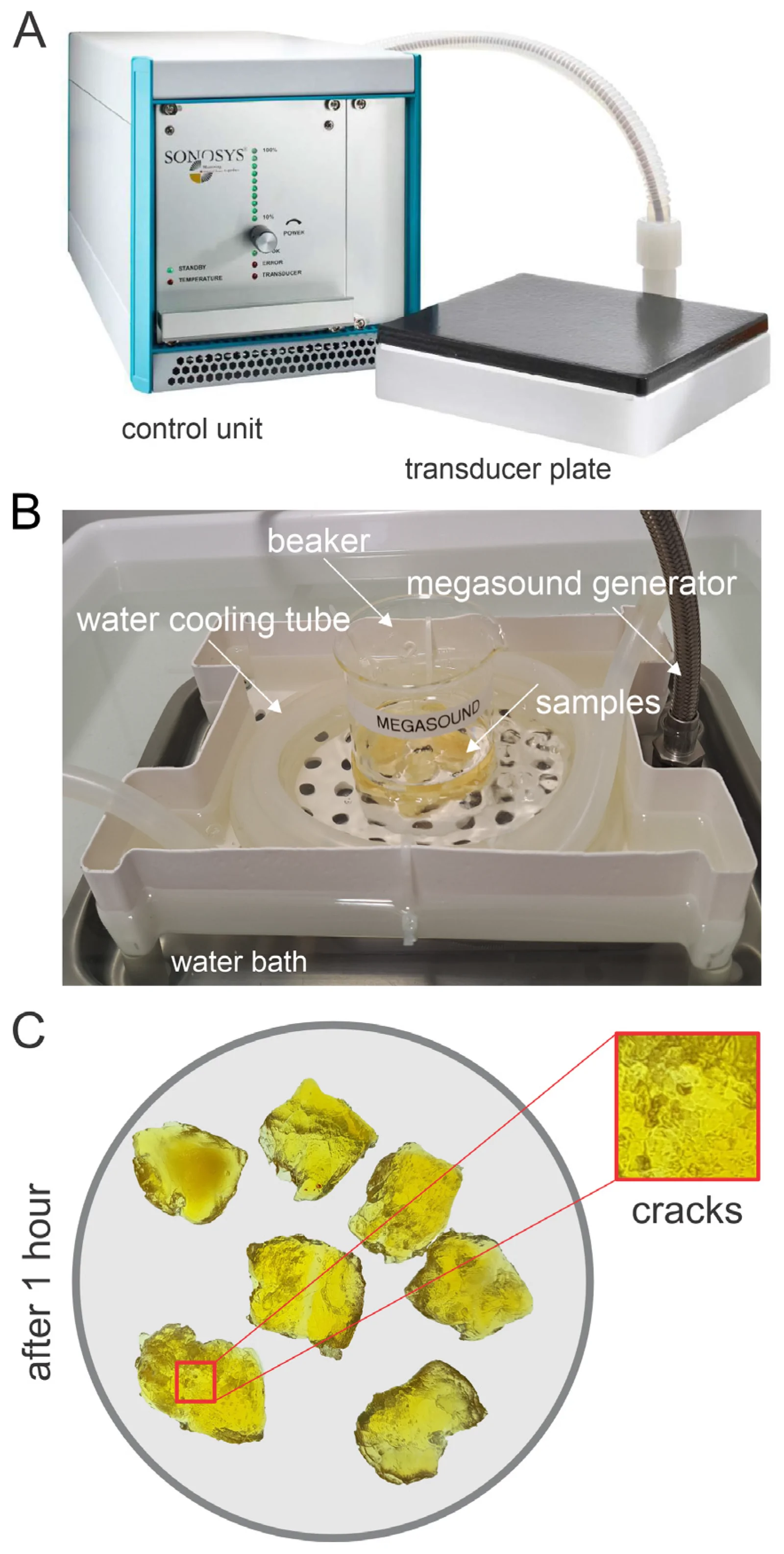
**The 1 MHz megasound system and induced artifacts.** (**A**) The 1 MHz megasound system consisting of a control unit and a transducer plate (Sonosys, Germany). (**B**) Experimental setup showing the sample beaker placed in a water bath above the megasound generator, cooled by a water tube. (**C**) Magnified view of tissue samples of porcine muscle samples after 1 h of 1 MHz treatment. Red boxes highlight the formation of cracks and micro-fissures in the tissue, an artifact specific to the high-frequency treatment.

For testing 1 MHz ultrasound, we utilized a Sonosys Megasound Generator (MHM-series, Sonosys, Neuenbürg, Germany) equipped with a 4-inch transducer system, providing a maximal ultrasound power of 500 W (Figure 2A). To ensure an energy transfer comparable with the output power of the 40 kHz ultrasound device, the 1 MHz Sonosys device was operated at 60% of its maximal power, i.e., at 300 W. According to the manufacturer’s calibration data, optimal energy transfer from the 1 MHz ultrasound bath into the immersed beakers required a glass wall thickness of approximately 3 mm. This condition was met using 250 mL standard beakers (Carl Roth, Karlsruhe, Germany). Similar to the 40 kHz setup, the temperature was maintained at a stable 20 °C using a comparable cooling system, supplemented with ice in the water bath.

**Spectroscopic Analysis:** To quantify optical transparency, cleared samples were placed in a high-precision optical glass cuvette (Hellma, Müllheim, Germany; 2 cm path length) filled with DBE. Transmission spectra were recorded in the visible range (400 to 700 nm) using a Hitachi U-5100 Spectrophotometer. (Hitachi, Vienna, Austria).

**Tissue Preparation and Dehydration:** (a) *Porcine muscle samples*: Tissue samples (*Musculus gracilis* and *Musculus pectineus*) were obtained from a local abattoir. The tissue was dissected into 12 cubic blocks of approximately 2 cm edge length and 10 larger pieces of about 4 to 5 cm in length and 2–3 cm width. The samples were fixed overnight in 500 mL of a 4 °C cold 4% formaldehyde solution. Following three wash cycles in 250 mL phosphate-buffered saline (PBS) for 5 min each, the samples were bleached for 90 min in 500 mL 2% hydrogen peroxide in PBS. Afterward, active chemical dehydration was performed with DMP (Merck, Vienna, Austria). Briefly, 12 tissue blocks and two larger pieces of tissue were immersed in 500 mL DMP in an Erlenmeyer flasks. Then the solution was acidified with 500 µL of 4 M HCl as a catalyst. After briefly shaking the spontaneous onset of the endothermic reaction, indicating the hydrolysis of DMP to methanol and acetone, was monitored via a temperature drop below 10 °C. The wide-mouth Erlenmeyer flask containing the samples was sealed with Parafilm to prevent evaporation and gently agitated on an orbital shaker. After 3 h, the solution was exchanged with fresh DMP, acidified again with HCL, and the samples were further incubated overnight to ensure complete dehydration.

(b) *Human auricles:* The cymba conchae of the auricles of five body donors, who voluntarily donated their dead bodies to the Division of Anatomy, Medical University of Vienna, for use in science and research, were extracted in compliance with institutional ethical guidelines in the Center of Anatomy and Cell Biology at the Medical University of Vienna (EK Nr. 1357/2024). They were fixed, bleached, and dehydrated following the protocol used for porcine muscle samples. However, processing was conducted separately for each sample in sealed snap-cap vials filled with ~5 mL of each solution. The vials were kept on an orbital shaker during processing. After 3 h the DMP was substituted with fresh acidified DBE (100 µL 4 N HCL per 100 mL). To minimize light scattering from lipids as myeline the samples were incubated after DMP dehydration for one hour in the potent organic lipid solver dichloromethane (5 mL per vial) (DCM; Merck, Darmstadt, Germany) [16].

**Refractive Index Matching and Ultrasound Treatment:** Refractive index matching was performed using dibenzyl ether (DBE; Merck, Darmstadt, Germany) (RI = 1.562). Prior to use, DBE was purified by column filtration over basic activated aluminum oxide (Brockmann Grade I, Merck, Darmstadt, Germany) to remove peroxides and benzaldehyde formed through contact with oxygen [17]. To ensure anhydrous conditions, a small amount (about a teaspoon per 100 mL) of water-free zeolite-based molecular sieve (3 Å pore size, Merck, Darmstadt, Germany) was added to the incubation vessels.

Of the 12 prepared small porcine samples, six were transferred to a 250 mL square, wide-neck bottle filled with 200 mL of fresh, peroxide-free DBE (Carl-Roth, Karlsruhe, Germany). This bottle was then immersed in a water-filled ultrasound bath (Emmi-D60, EMAG, Germany; 40 kHz, 320 W) equipped with a cooling system (ultrasound group) (Figure 1). The remaining six small samples were incubated in a second, identical flask containing 250 mL of DBE on an orbital shaker at room temperature (control group). It is crucial that the flasks are firmly sealed with a lid to prevent the ingress of moisture. We also recommend using rectangular flasks, as they better optimize the available space inside the ultrasound bath. Specifically, flasks measuring 64 mm in depth and 64 mm in width proved to be the optimal fit for our Emmi-D60 ultrasound bath.

Of the 10 dehydrated larger porcine samples, five were assigned to the control group and five to the ultrasound treatment group. Due to their larger size, the samples for each group were distributed across two bottles, with each bottle containing 200 mL of DBE. The treatment duration was optimized between 1 and 4 h, depending on the sample size. For the time-course analysis of the clearing process, photographic documentation of the samples was performed every 60 min. Following ultrasound treatment, the samples were transferred to fresh DBE for long-term storage.

**Light Sheet Microscopy:** For assessing the clearing quality at the microscopic level the optically cleared human auricle samples were imaged utilizing a meso-aspheric-based light sheet microscope [18,19]. To elucidate the tissue’s structural composition, triple-excitation autofluorescence imaging (at wavelengths of 488 nm, 532 nm, and 640 nm) was employed.

## 3. Results

**Figure 3:**
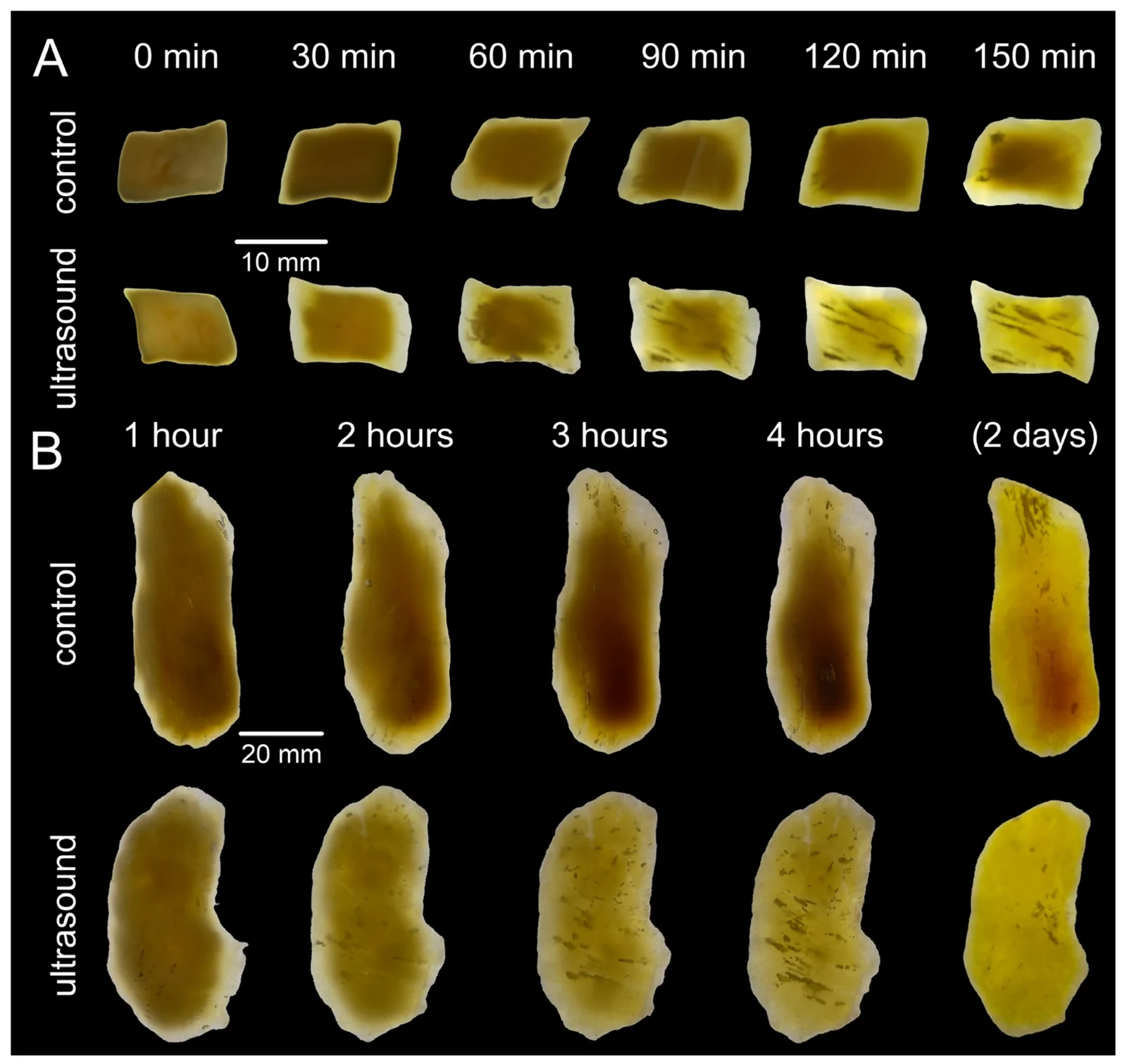
**Acceleration of clearing kinetics in porcine muscle tissue.** (**A**) Time-course comparison of porcine muscle blocks cleared passively (left panel) versus those treated with 40 kHz ultrasound (right panel). While control samples show only marginal clearing over 150 min, the ultrasound-treated samples exhibit rapid transparency, becoming nearly transparent within 90 min. (**B**) The acceleration becomes even more pronounced with larger samples of multiple centimeter size.

We observed that ultrasound treatment clearly facilitated the clearing process. Porcine muscle samples treated with 40 kHz ultrasound were noticeably more transparent at their edges after 1 h of treatment, clearly demonstrating the enhanced diffusion speed of the clearing medium under acoustic processing (Figure 3).

**Figure 4:**
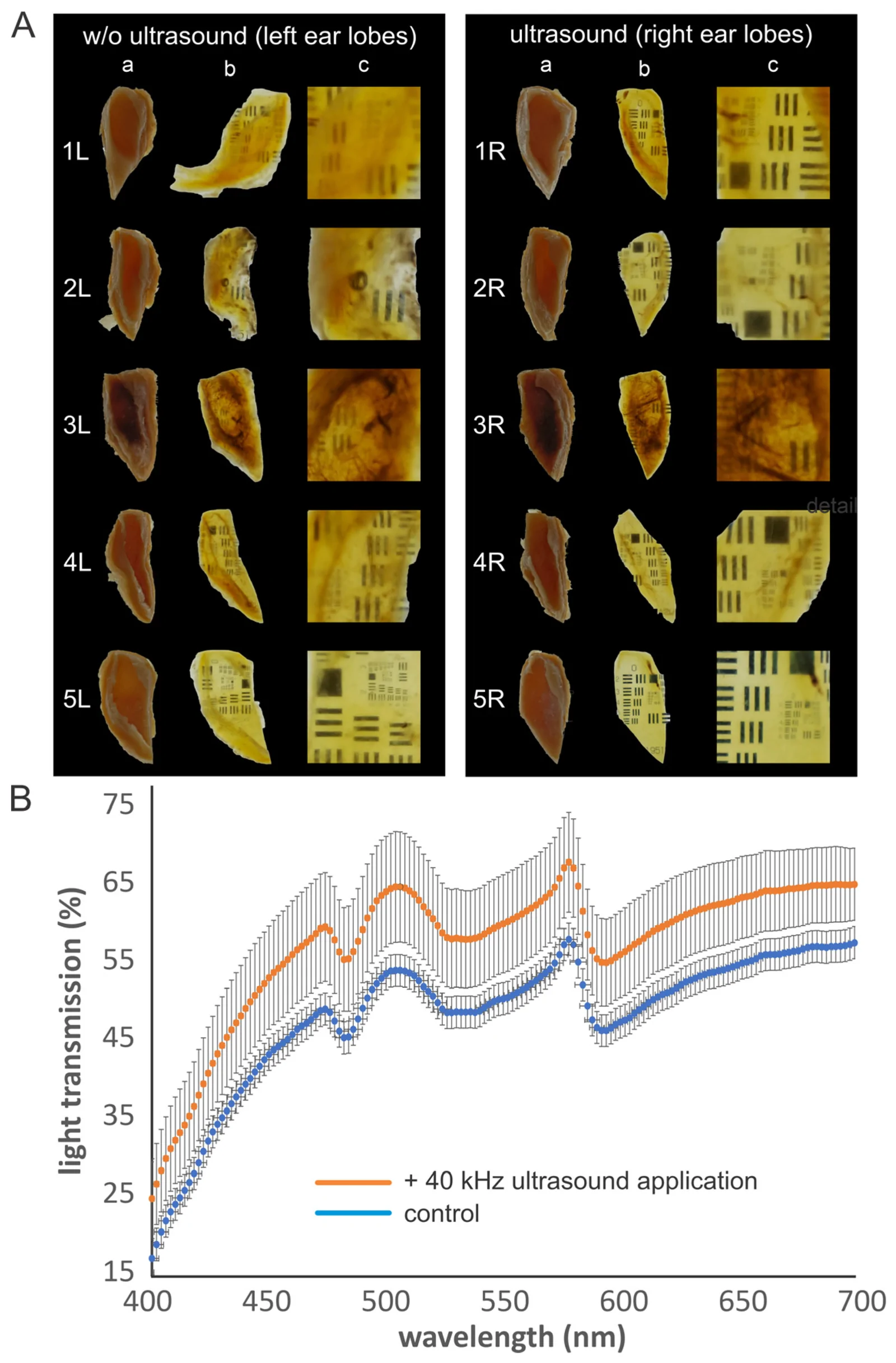
**Long-term stability and quantification of transparency in human ear samples.** (**A**) Comparison after one year of storage in DBE. The ultrasound-treated samples (**right panel**) remain visibly clearer than standard controls (**left panel**). (**a**) Uncleared samples. (**b**) Cleared samples. (**c**) Detail view of (**b**). For better assessment of transparency the samples were placed on an USAF test chart. (**B**) Transmission spectroscopy (400–700 nm) quantifies the improvement: ultrasound-treated samples (orange line) show significantly higher light transmission percentages compared to controls (blue line), confirming superior optical clarity even after one year of storage in DBE.

Similarly, the human ear samples exhibited a comparable acceleration in clearing speed (Figure 4A). Remarkably, this enhanced transparency proved highly stable over time. Spectroscopic quantification performed after one year of storage in DBE revealed that the ultrasound-treated specimens maintained significantly higher light transmission across the visible spectrum compared to the passively cleared controls (Figure 4B).

**Figure 5:**
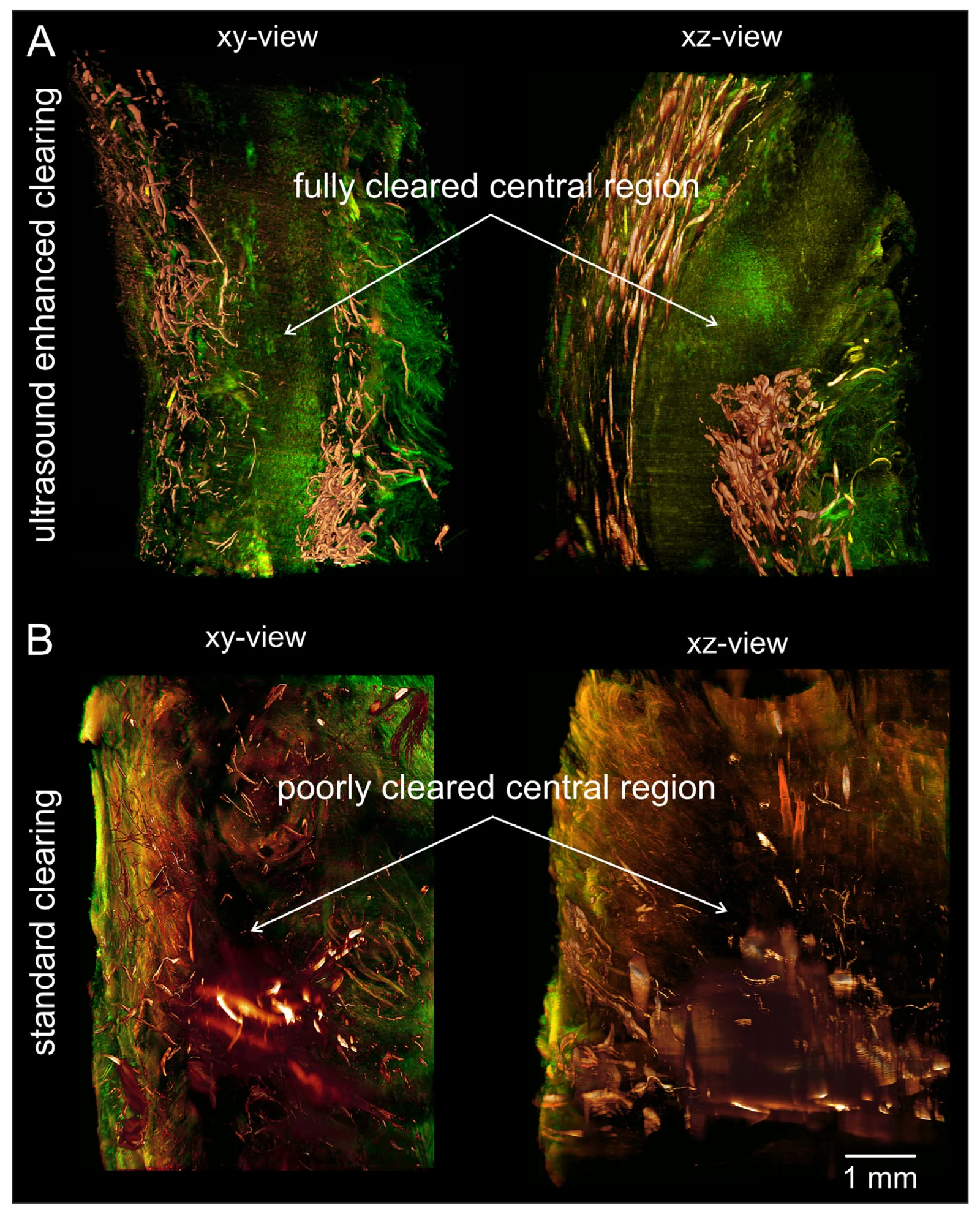
**Enhanced 3D imaging quality of ear samples verified by light sheet microscopy.** (**A**) 3D reconstruction of a human ear biopsy prepared with ultrasound-enhanced clearing. The central region is fully cleared, allowing for sharp visualization of deep tissue structures without scattering artifacts. (**B**) 3D reconstruction samples cleared without ultrasound treatment. The XY and orthogonal XZ views reveal a poorly cleared central region with high light scattering. Autofluorescence imaging was conducted using excitation wavelengths of 488 nm, 532 nm, and 640 nm; scale bar: 1 mm.

Light sheet microscopy confirmed that the earlobe samples subjected to ultrasound-assisted clearing displayed greater detail and substantially enhanced sharpness deep within the tissue. This ultrasound-assisted protocol yielded a fully transparent central region, enabling the 3D reconstruction of the entire tissue volume with less artifacts (Figure 5A). In contrast, standard passive clearing resulted in a poorly cleared central region characterized by significantly higher light scattering and a distinct loss of spatial resolution (Figure 5B).

## 4. Discussion

In comparative experiments, we additionally investigated the effect of higher-frequency ultrasound waves using a 1 MHz “megasound” generator provided by Sonosys, Germany. We found that this high-frequency treatment yielded no advantage over the standard 40 kHz frequency in terms of clearing speed or quality. On the contrary, 1 MHz irradiation at comparable energy levels frequently induced macroscopic damage, specifically micro-fractures within the tissue matrix (Figure 2C). These artifacts were entirely absent in samples treated at 40 kHz.

This frequency-dependent difference in the preservation of tissue integrity may be explained by the nature of ultrasound’s mechanical effects, principally cavitation [20]. The likelihood of cavitation occurrence can be estimated by the mechanical index (*MI*), defined as the peak negative pressure (*P_NP_*) divided by the square root of the center frequency *f_c_*, expressed as

$$MI=\frac{P_{NP}}{\sqrt{f_{c}}}$$

where *MI*: mechanical index, *P_NP_*: peak negative pressure, and *f_c_*: center frequency [21]. Assuming an approximately equal acoustic power of 300 W for both devices, the peak negative pressures *P_NP_* generated by each system can be considered roughly equivalent. Therefore, the ratio of their mechanical indices depends primarily on the frequency difference. By comparing the 40 kHz (*f_c_* = 0.04 MHz) and 1 MHz (*f_c_* = 1 MHz) systems, the calculation unfolds as

$$\frac{MI_{40kHz}}{MI_{1MHz}}=\frac{P_{NP}/\sqrt{0.04}}{P_{NP}/\sqrt{1}}=5,$$

suggesting that the 40 kHz system generates an *MI* about 5 times higher than the 1 MHz system. Although it may be expected that a higher *MI* is associated with a greater risk of inertial cavitation and subsequent tissue damage, the physical dynamics shift significantly at the low frequencies characteristic of a standard laboratory ultrasound bath. At 40 kHz, the longer acoustic waves give bubbles ample time to grow and oscillate steadily. Thus, we propose that the 40 kHz system induces “stable” (non-inertial) cavitation, despite its higher calculated MI. This more stable cavitation creates acoustic microstreaming, a steady fluid flow in the boundary layer of oscillating microbubbles [21]. This microstreaming likely generates high local shear stress that effectively disrupts the stagnant diffusion boundary layers within the dense tissue matrix, thereby actively forcing the viscous clearing agent into the sample without causing damage. In contrast, the 1 MHz system generates rapid pressure oscillations. While it possesses a lower calculated MI, these rapid cycles afford bubbles very little time to grow before forcing them into a violent collapse, resulting in inertial cavitation. This more aggressive energy release produces intense localized shockwaves that physically tear the dense tissue matrix, directly explaining the micro-fractures and reduced tissue integrity observed with the 1 MHz system.

Our results reveal a significant practical finding: a standard, inexpensive 40 kHz ultrasound bath can be used to speed up the clearing procedure and markedly improve the final transparency of solvent-based tissue clearing. A key advantage of this approach is the broad accessibility of the equipment. While specialized laboratory sonication devices can be costly, standard ultrasonic cleaners, such as the unit used in this study (approx. 320 W), are ubiquitous in laboratories for cleaning mechanical or optical components and are available for a fraction of the cost (<$1000) of specialized histology equipment. This lowers the barrier to adopting high-quality tissue clearing of large specimens such as tumor biopsies or human brain samples in standard research settings. We specifically contrasted this accessible technology with high-frequency “megasound” (1 MHz) generators, which are typically employed in the semiconductor industry for precision cleaning and are significantly more costly (typically > $20,000). Our data show that this increased investment does not yield better clearing results. Instead, at comparable energy inputs, 1 MHz ultrasound tended to damage tissue integrity and induce micro-fractures.

While our study establishes a rapid and accessible baseline protocol for ultrasound-enhanced tissue clearing using standard laboratory equipment, several limitations warrant consideration for future research. First, our primary objective was to demonstrate the fundamental physical viability of the 40 kHz ultrasound protocol on healthy tissues. As diseased clinical specimens and advanced organoid models exhibit significantly higher heterogeneity and diffusion resistance, it remains the responsibility of individual researchers to verify and adapt this method for highly complex pathological conditions. Second, to isolate physical clearing efficacy and assess structural integrity without the confounding variables of antibody penetration kinetics, we relied on autofluorescence imaging. The compatibility of this method with specific immunolabeling protocols has not yet been evaluated and requires separate, dedicated validation. Third, to maintain broad accessibility, we tested fixed, standard parameters (40 kHz/320 W and 1 MHz/300 W) characteristic of ubiquitous laboratory cleaners. We cannot exclude the possibility that different frequency and power settings might provide better results for various types of tissue. However, testing this would require a research-grade ultrasound device that allows for frequency and power adjustments over a broad range. Such devices require an investment of thousands of dollars. The goal of this publication was to provide a cost-efficient and accessible method to enhance tissue clearing with easily accessible standard laboratory equipment. Therefore, while our parameters serve as an effective baseline, individual laboratories will need to perform specific optimizations for tissues of varying sizes and morphologies. Finally, our tissue damage evaluation focused on macroscopic and microscopic structural integrity to address the immediate risk of physical micro-fractures. We did not conduct molecular-level assays to assess the potential degradation of intrinsic proteins, nucleic acids, or lipids. Researchers intending to perform sensitive downstream molecular assays must independently evaluate biomacromolecule preservation following ultrasound treatment.

## 5. Conclusions

We demonstrated that standard ultrasonic baths, typically used for equipment degreasing and cleaning, can be efficiently repurposed to enhance the speed and outcome of organic tissue clearing. The application of 40 kHz ultrasound, a standard operating frequency for these devices, offers a highly accessible and cost-effective method to accelerate the refractive index matching step in chemical tissue clearing. This approach is particularly advantageous for large samples, effectively overcoming the method’s temporal bottleneck.

## Data Availability

All data and images are available on request from the authors.

## Abbreviations

The following abbreviations are used in this manuscript:

DBE	Dibenzyl ether
DCM	Dichloromethane
DMP	2,2-dimethoxypropane
HCL	Hydrochloric acid
LSFM	Light sheet fluorescence microscopy
MI	Mechanical index
PBS	Phosphate-buffered saline
RI	Refractive index
